# Supplementary figures and images for: Energy-efficient and reliable dual closed-loop DC control system for intelligent electric vehicle charging infrastructure
Source: PLoS One. 2024 Dec 16;19(12):e0315363. doi: 10.1371/journal.pone.0315363 (PMC11649101; doi:10.1371/journal.pone.0315363)

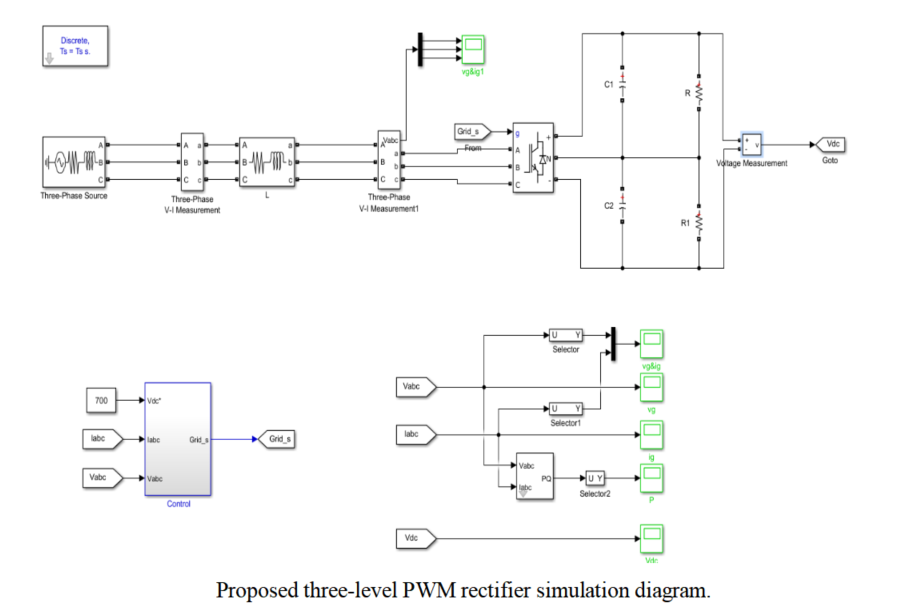

Supplement: S1 Fig — (TIF) [file pone.0315363.s001.tif]

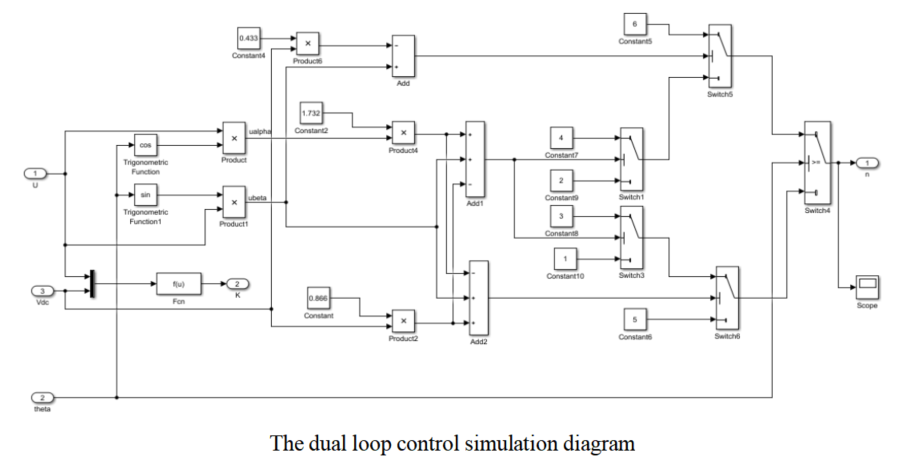

Supplement: S2 Fig — (TIF) [file pone.0315363.s002.tif]
